# Incarcerated giant uterine leiomyoma within an incisional hernia: a case report

**DOI:** 10.1002/ccr3.1199

**Published:** 2017-09-26

**Authors:** Georgios Exarchos, Nikolaos Vlahos, Dionysios Dellaportas, Linda Metaxa, Theodosios Theodosopoulos

**Affiliations:** ^1^ 2nd Department of Surgery Aretaieion University Hospital Athens Greece; ^2^ Department of Obstetrics and Gynecology Aretaieion University Hospital Athens Greece; ^3^ Radiology Department St Bartholomew's Hospital London UK

**Keywords:** Fibroids, incarcerated leiomyoma, incisional hernia, ventral abdominal wall hernia

## Abstract

Uterine leiomyomas presenting as incarcerated or strangulated hernias in surgical emergencies are extremely rare and should be considered in the differential diagnosis in patients with known uterine fibroids and an irreducible ventral abdominal wall hernia. Detailed history and multidisciplinary approach optimize the diagnosis and decision making toward surgical treatment.

## Introduction

Uterine leiomyomas (also known as fibroid tumors) are the commonest benign uterine lesions and can be histopathologically revealed in up to 70% of patients undergoing hysterectomy 1. Giant uterine leiomyomas are unusual 2 and can produce several symptoms and discomfort to the patient.

A midline abdominal incision is the commonest incision used in open general surgery procedures. It is mostly favorable because surgeons achieve access to the abdominal cavity with limited damage to the muscles, nerves, and blood supply of the abdominal wall, while it is quick and easy to perform 3. However, incisional hernias can occur in about 10% of the cases 1 as long‐term incidence causing in most cases discomfort and pain as parts of the abdominal viscera can be trapped in the hernia sac. The commonest contents of the sac in an incisional ventral abdominal wall hernias are adipose tissue, part of the small bowel, the transverse colon, or omentum, as these organs are freely movable in the peritoneal cavity 1. Unusual hernia sac contents can be occasionally encountered and surgical experience, and careful dissection is of paramount importance in decision making and avoidance of complications.

However, an incarcerated giant leiomyoma within an incisional hernia is a very rare entity, surprising the surgeon, and raising multiple management queries 4, 5, 6, 7.

A rare case of a giant uterine leiomyoma incarcerated into an incisional hernia is presented here, and management options and concerns are highlighted.

## Case Presentation

A 56‐year‐old, nulliparous woman, with previous surgical history of an open paraumbilical hernia repair without the use of prosthetic mesh a year ago was referred due to diffuse abdominal pain. The patient was on antipsychotic medication for schizophrenia, a heavy current smoker (30 pack/years) and morbidly obese (body mass index 35). Patient's mental capacity was assessed using the legally relevant criteria, and she was found to be incompetent. Therefore, her brother acted as decision maker and consented for further investigation and treatment.

The patient had normal pulse (85/min) and arterial blood pressure (130/82 mmHg), remaining apyrexial. She was initially assessed by the surgical team, and she was found to have an approximately 4‐cm irreducible incisional paraumbilical hernia. The content and texture of the hernia sac were round and firm clinically. Tenderness was elicited with deep palpation, and an incarcerated incisional hernia was the initial working diagnosis. Routine hematological and biochemical laboratory investigations were unremarkable, and abdominal X‐ray did not show any signs of small bowel obstruction. Lactate levels were within normal range.

Digital rectal examination identified a large painful pelvic mass and no signs of bleeding. A gynecological examination was requested, but there were limitations for further assessment as the patient was virgin, and she could not undergo a per‐vagina examination. Considering all the above findings and body habitus, an urgent abdominal CT scan was performed. The latter showed a 15 cm solid pelvic mass with scattered calcifications originating from the uterus or adnexa suggesting a large uterine leiomyoma. The mass was extending up to the level of a sizable lower abdominal midline incisional hernia. The hernia sac was filled with small bowel loops on imaging. No free fluid or pneumoperitoneum was noted. The patient was discussed in our MDT with gynecological input, and a decision was taken to proceed with an exploratory laparotomy.

Of note, she was postmenopausal and nulliparous, and a decision had to be made regarding the potential scenario of hysterectomy. Following an extensive discussion with her brother, consent was obtained to proceed with hysterectomy if needed.

As a joint care with the Gynecologists, surgical exploration was decided and performed via a midline laparotomy incision along the previous abdominal wall scar. Cefuroxime 1.5 g was given at anesthetic induction for antimicrobial prophylaxis.

**Figure 1:**
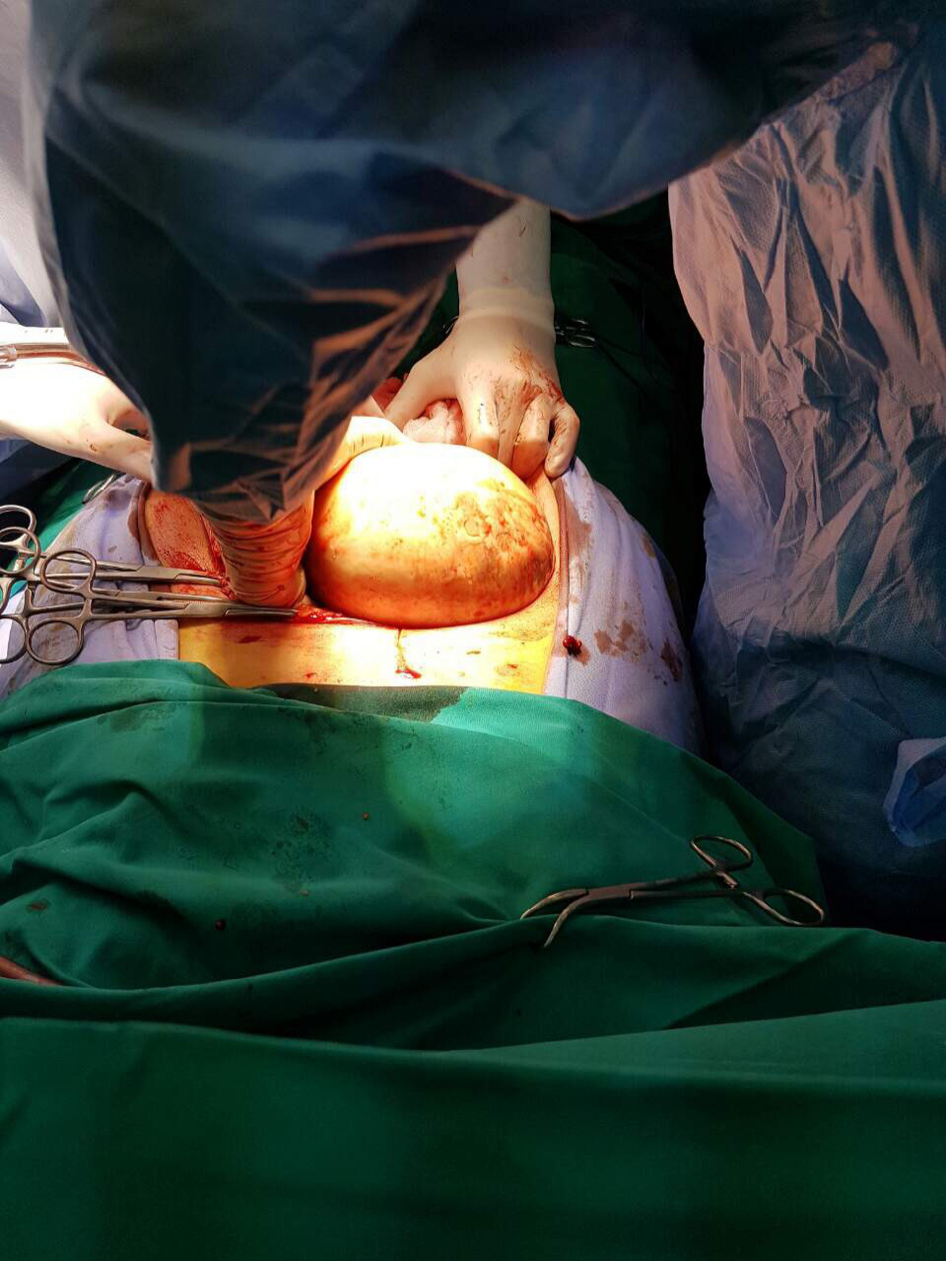
A large incarcerated leiomyoma seen within the hernia sac following midline laparotomy incision.

**Figure 2:**
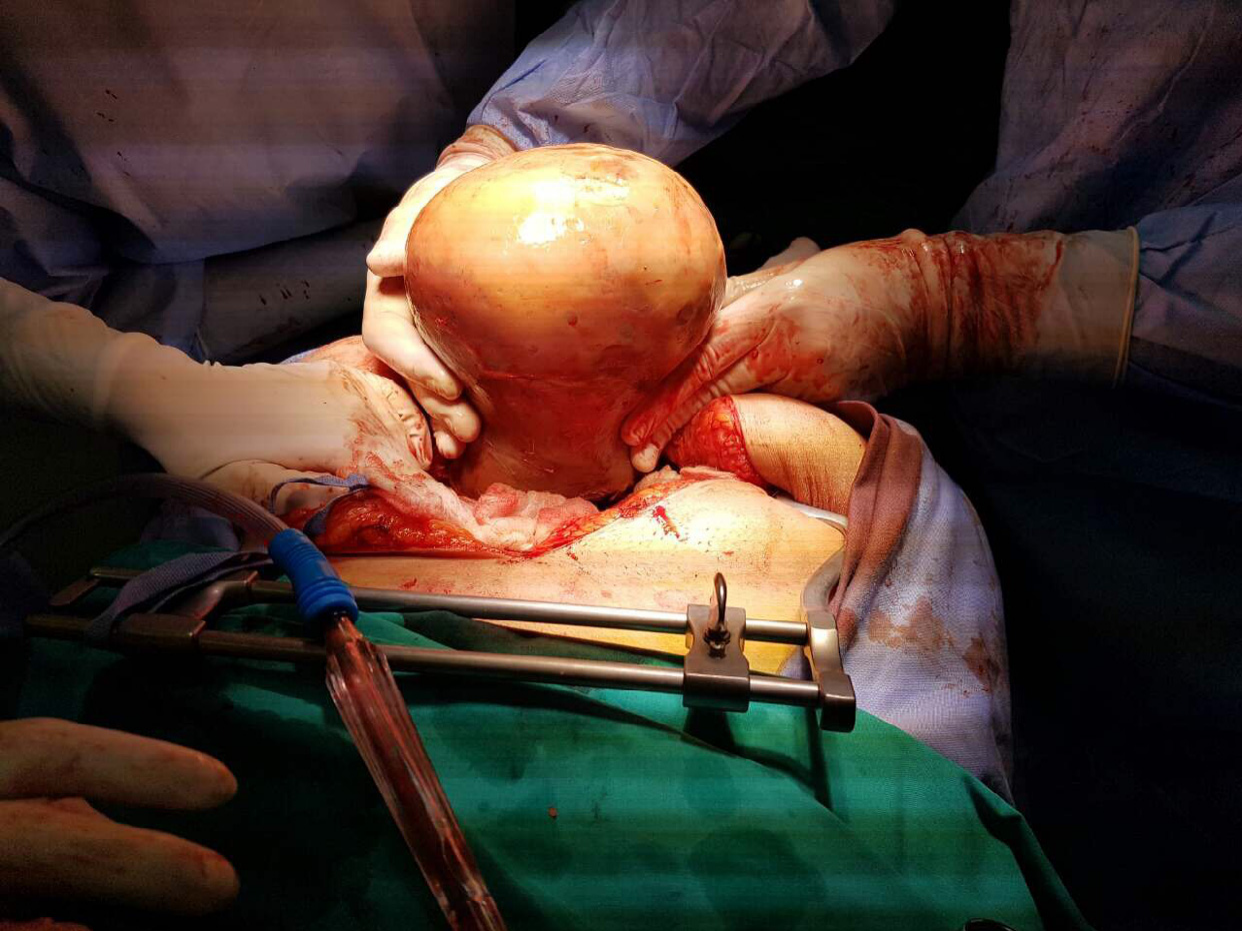
The large leiomyoma was almost completely into the hernia sac and incarcerated, while the stalk of the tumor was occupying the neck of the hernia defect.

Surprisingly, and against CT scan findings, a large incarcerated solid mass was seen within the hernia sac (Fig. 1). There was no small bowel protruding through the abdominal wall defect. The large mass lesion was almost completely into the hernia sac and incarcerated, while the stalk of the tumor was occupying the neck of the hernia defect (Fig. 2). Further exploration revealed the origin of this lesion being the uterus, and therefore, total abdominal hysterectomy and bilateral salpingoophorectomy were decided and performed by a Consultant Gynecologist. The incisional hernia was primarily repaired by the Consultant General Surgeon, using no prosthetic mesh, due to the emergent operative setting and infective contamination concerns. A negative pressure drain was inserted above the repaired hernia defect to prevent a seroma formation.

**Figure 3:**
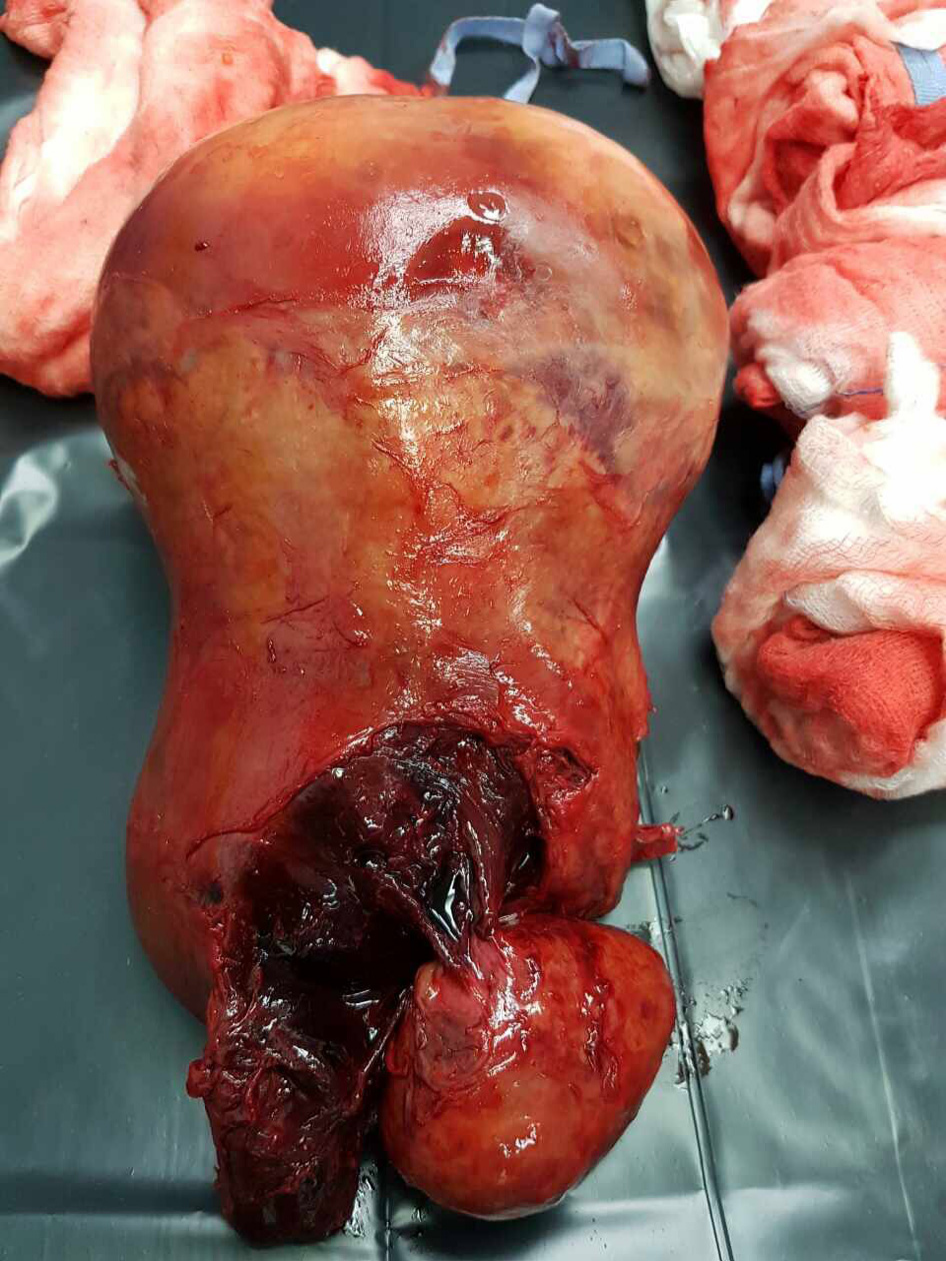
Final histopathology of the tumor showed a 19 × 14 × 9 cm uterine leiomyoma with ischemic and hemorrhagic necrosis, calcifications and hyalinization with no evidence of dysplasia or malignancy.

Final histopathology of the tumor showed a 19 × 14 × 9 cm uterine leiomyoma with ischemic and hemorrhagic necrosis, calcifications, and hyalinization with no evidence of dysplasia or malignancy (Fig. 3).

The patient had an uneventful postoperative course, and standard antipsychotic medication was promptly restarted. On the second postoperative day, she accidentally pulled her negative pressure drain off, and a small seroma was formed, which was gradually absorbed. She was discharged on the 7th postop day.

## Discussion

Incisional umbilical and paraumbilical hernias, described for the first time back in 1968, are unusual entities, with an incidence of 21/100,000 in the Mintz study 8. Predisposing factors for incisional hernia development are wound infection, obesity, age, diabetes mellitus, prolonged coughing due to smoking or long‐standing lung infections and incisions more than 18 cm 1. In general, midline ventral abdominal wall hernias contain adipose tissue, small bowel loops, part of the transverse colon, or omentum, as these organs are more freely movable in the peritoneal cavity. In the current surgical literature, several cases of unusual contents found incarcerated in the hernia sac have been reported, such as the ovaries 9 and ovarian tumors 10, hepatic ligaments 8, urinary bladder 11, and gravid uterus 4, 5, 6, 7, 12, 13, 14, 15, 16, 17, 18 with/without leiomyomas. Round ligament leiomyomas have been reported within inguinal hernia sacs 19, 20, 21, 22. To the best of our knowledge, only five cases are reported in the English medical literature, associated with incarcerated leiomyomas in an umbilical hernia sac, four of which involving pregnant women 4, 5, 6, 7. This case is the second of an incarcerated leiomyoma in a nongravid uterus within an incisional hernia; however, in the current case, the leiomyoma was giant, and presentation was acute, requiring emergency surgery. Previous midline laparotomy, obesity, chronic constipation, and smoking predisposed to the incisional hernia, which enlarged over time. The large leiomyoma contributed to the increased intra‐abdominal pressure and protrusion of abdominal viscera into the hernia sac. Obviously, small bowel loops and the large leiomyoma were alternating into the hernia sac, causing intermittent abdominal pain and discomfort. As reported by Fehintola et al. 23, the mechanism of incarceration of fibroids in a nonpregnant uterus is difficult to explain and the probable pathophysiological scenario is that the huge leiomyoma increased the intra‐abdominal pressure and caused incarceration of abdominal viscera into the hernia. Large smooth masses in women, in the absence of history of known malignancy, can be originating from the female genitalia. Before embarking to surgical exploration, this possibility should be highlighted to the patient and consider hysterectomy and salpingo‐oophorectomy when consenting patients is prudent. Despite imaging accuracy, complex cases like the one presented above can confuse even very experienced senior surgeons.

## Conclusion

Uterine leiomyomas presenting as incarcerated or strangulated hernias in surgical emergencies are extremely rare and should be considered in the differential diagnosis in patients with known uterine fibroids and an irreducible ventral abdominal wall hernia. Detailed history and meticulous clinical examination in cooperation with other surgical disciplines when required as well as radiological evaluation with ultrasound scan and computed tomography are important adjunctive tools of a multidisciplinary team optimizing the diagnosis and decision making toward surgical treatment. In an era of extremely accurate imaging modalities, surgery can still reveal unexpected findings and requires high level of experience and teamwork to deal with complex clinical scenarios.

## Authorship

GE: was the main author, wrote and reviewed manuscript and performed a literature review. NV: Provided the intra‐operative figures and the gynecological input in patient's management. DD: Contributed in edition of final manuscript. LM: Reviewed patient's CT imaging, contributed to study design and performed a literature review. TT: Involved in overall description of the manuscript and provided the intra‐operative pictures.

## Conflict of Interest

None declared.
